# CTCF-KDM4A complex correlates with histone modifications that negatively regulate *CHD5* gene expression in cancer cell lines

**DOI:** 10.18632/oncotarget.24798

**Published:** 2018-03-30

**Authors:** Lissania Guerra-Calderas, Rodrigo González-Barrios, Carlos César Patiño, Nicolás Alcaraz, Marisol Salgado-Albarrán, David Cantú de León, Clementina Castro Hernández, Yesennia Sánchez-Pérez, Héctor Aquiles Maldonado-Martínez, Inti A. De la Rosa-Velazquez, Fernanda Vargas-Romero, Luis A. Herrera, Alejandro García-Carrancá, Ernesto Soto-Reyes

**Affiliations:** ^1^ Cancer Biomedical Research Unit, Instituto Nacional de Cancerología (INCan), Mexico City, Mexico; ^2^ Instituto de Investigaciones Biomédicas, Universidad Nacional Autónoma de México (UNAM), Mexico City, Mexico; ^3^ Clinical Research, Instituto Nacional de Cancerología (INCan), Mexico City, Mexico; ^4^ The Bioinformatics Centre, Section for RNA and Computational Biology, Department of Biology, University of Copenhagen, Copenhagen, Denmark; ^5^ Department of Surgical Pathology, Instituto Nacional de Cancerología, Mexico City, Mexico; ^6^ Genomics Lab, Universidad Nacional Autónoma de México, Red de Apoyo a la Investigación-CIC and Instituto Nacional de Ciencias Médicas y Nutrición “Salvador Zubirán”, Mexico City, Mexico; ^7^ Instituto de Fisiologia Celular-Neurociencias, Universidad Nacional Autonoma de Mexico (UNAM), Mexico City, Mexico

**Keywords:** KDM4A, CTCF, histone demethylation, H3K36me, CHD5

## Abstract

Histone demethylase KDM4A is involved in H3K9me3 and H3K36me3 demethylation, which are epigenetic modifications associated with gene silencing and RNA Polymerase II elongation, respectively. *KDM4A* is abnormally expressed in cancer, affecting the expression of multiple targets, such as the *CHD5* gene. This enzyme localizes at the first intron of *CHD5*, and the dissociation of KDM4A increases gene expression. *In vitro* assays showed that KDM4A-mediated demethylation is enhanced in the presence of CTCF, suggesting that CTCF could increase its enzymatic activity *in vivo,* however the specific mechanism by which *CTCF* and *KDM4A* might be involved in the *CHD5* gene repression is poorly understood. Here, we show that CTCF and KDM4A form a protein complex, which is recruited into the first intron of *CHD5*. This is related to a decrease in H3K36me3/2 histone marks and is associated with its transcriptional downregulation. Depletion of *CTCF* or KDM4A by siRNA, triggered the reactivation of *CHD5* expression, suggesting that both proteins are involved in the negative regulation of this gene. Furthermore, the knockout of *KDM4A* restored the *CHD5* expression and H3K36me3 and H3K36me2 histone marks. Such mechanism acts independently of *CHD5* promoter DNA methylation. Our findings support a novel mechanism of epigenetic repression at the gene body that does not involve promoter silencing.

## INTRODUCTION

Gene regulation in eukaryotes is driven in part by chromatin architecture, where histone post-translational modifications play a major role in this process [1]. In particular, the methylation of lysine residues in histones is involved in transcriptional activation and repression, depending on specific lysines and the degree of methylation. For example, H3K4me3 and H3K36me3 are associated with transcriptional activation, while H3K9me3 and H3K27me3 are related with transcriptional repression [2].

Although, it was long thought that lysine methylation was a stable and irreversible process, recent reports have found approximately 25 enzymes capable of removing the methyl groups of lysines in histones. These enzymes are grouped into two families depending on their chemical mechanism of demethylation, the oxidases and the oxygenases [3]. The majority of histone demethylases belong to the second family, including lysine (K)-specific demethylase 4A (KDM4A). KDM4A actively removes the methyl groups from H3K36me3 to produce H3K36me2 [3]. In particular, H3K36me3 is enriched in genes that are transcriptionally active and is associated with recruitment of RNA polymerase II and transcriptional elongation, loss of H3K36me3 leads to transcriptional repression [4].

*KDM4A* is overexpressed in several types of cancer, including breast cancer [5]. One of the target genes of KDM4A is chromodomain helicase DNA binding protein 5 gene (*CHD5*). *CHD5* was identified as a tumor suppressor gene, and it has been reported deregulated in glioma, colon, lung, ovarian, prostate and breast cancers. Thus, based on its likely involvement as a tumor suppressor gene (TSG) in neuroblastomas, gliomas, and many common adult neoplasms, CHD5 may play an important developmental role in many other tissues besides the nervous system and testis [6]. Particularly, this gene is involved in cell proliferation, apoptosis and senescence by regulating p19^Arf^, modulating p53 activity [6]. KDM4A has been reported to negatively regulate *CHD5* by its recruitment to the first intron [7]. Neither the mechanism by which KDM4A negatively regulates *CHD5* nor the mechanism by which KDM4A is recruited to this target site are known. Furthermore, *in vitro* assays have shown that the demethylation frequency of KDM4A increases up to 80% in the presence of the architectural protein CTCF [8], suggesting that CTCF may play a major role in the activity of KDM4A which has not been addressed until now. Hence, the aim of this study was to elucidate the mechanism underlying the role of CTCF and KDM4A on histone modifications and in the downregulation of *CHD5*.

## RESULTS

### *KDM4A* is highly expressed in MCF7, MDA-MB-231 and HeLa cell lines

**Figure 1:**
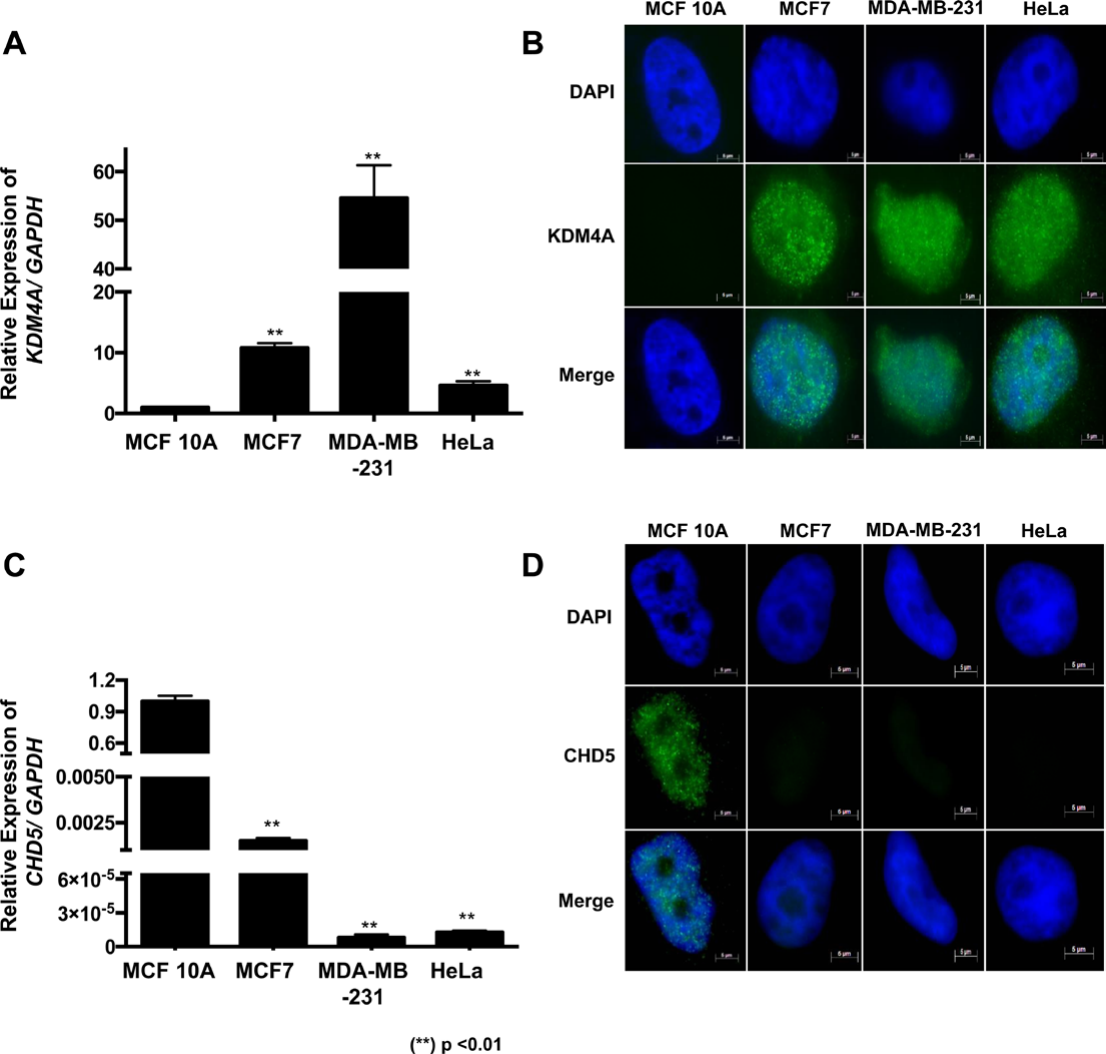
KDM4A overexpression correlates with CHD5 decrease in neoplastic cell lines (**A**) Expression profile of the human *KDM4A* gene in MCF 10A, MCF7, MDA-MB-231 and HeLa cell lines obtained by RT–qPCR. The data were normalized against GAPDH expression in three independent experiments. (**B**) The presence and localization of KDM4A in MCF 10A, MCF7, MDA-MB-231 and HeLa cells were assessed by immunofluorescence assay. (**C**) Expression profile of *CHD5* gene in the MCF 10A, MCF7, MDA-MB-231 and HeLa cell lines obtained by RT–qPCR. The data were normalized against GAPDH expression in three independent experiments. (**D**) The presence and localization of *CHD5* in MCF 10A, MCF7, MDA-MB-231 and HeLa cells were assessed by immunofluorescence assay. The DNA was stained with DAPI. (^**^) *p <* 0.01 compared with the MCF 10A cell line. Statistical differences were determined using Student's *t* test.

As a first approach, we evaluated the expression of *KDM4A* in four different cell lines using RT-qPCR. We observed that *KDM4A* was highly expressed in MCF7 and MDA-MB-231 cell lines compared to the expression levels of the non-tumorigenic epithelial breast cell line MCF 10A (Figure 1A). Previously, *KDM4A* has been reported to be highly expressed in HeLa cells [9], hence we used this cell line as a positive control. Immunofluorescence assays show that KDM4A is located mainly at the nucleus in the neoplastic cell lines (Figure 1B), but it is not detected in the non-tumorigenic breast cell line MCF 10A (Figure 1B). We also observed *CHD5*, which has been reported to be regulated by KDM4A and highly expressed in the MCF 10A cells compared with MCF7, MDA-MB-231 and HeLa cells (Figure 1C) [7]. Additionally, *CHD5* is only detected in the MCF 10A cell line, where *KDM4A* is not present (Figure 1B and 1D). When looking into breast cancer cell line expression data available at the Cancer Cell Line Encyclopedia we found that 83.34% (50/60) of these cell lines show high expression of *KDM4A,* while not expressing *CHD5.* In this regard, MCF7 and MDA-MB-231 cell lines exhibit the same behavior that we observed previously in our results (Figure 1 and Supplementary Figure 1A) [10]. In contrast to what is observed in cell lines, we did not find a significant correlation between *KDM4A* and *CHD5* expression in breast cancer patients (Supplementary Figure 1B) from The Cancer Genome Atlas (TCGA). We argue that this could be due to the heterogeneity of the tumor tissue or tumor subtypes.

### DNA methylation at the *CHD5* gene promoter is not the main mechanism of epigenetic silencing in the neoplastic cell lines

**Figure 2:**
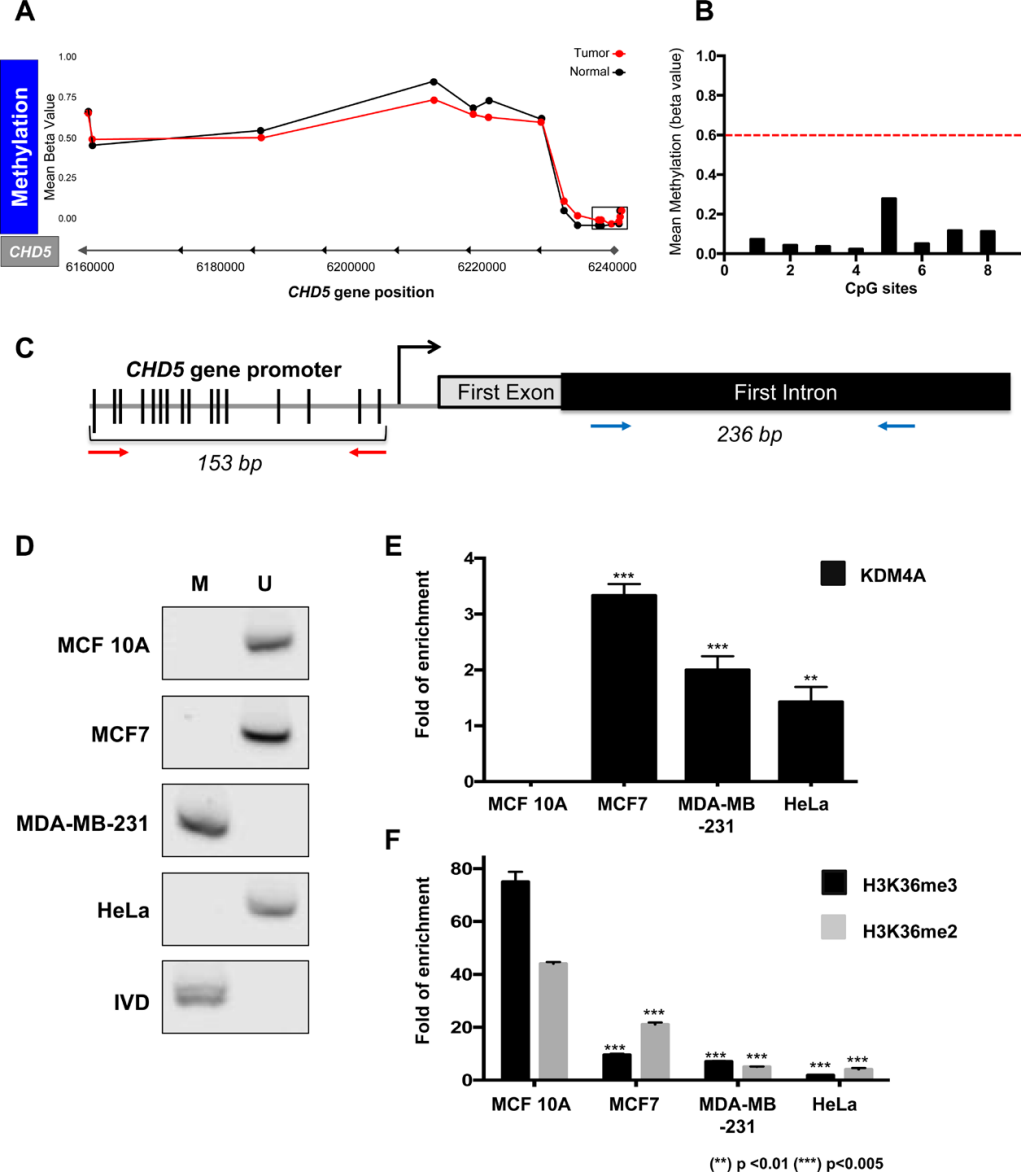
CHD5 repression is associated to histone demethylation by KDM4A at the first intron and not to promoter DNA methylation (**A**) TCGA DNA methylation levels (Ilumina 450 K data) in 743 breast cancer patients (tumor) and 98 non-neoplastic samples (normal) in CHD5 gene locus. 63 CpG sites were analyzed along the gene. Cutoff ≥0.6 Beta-values represents methylated status. The locus marked by a rectangle represents the methylation status of the promoter region; X axes represents the *CHD5* gene position (GRCh 37/hg19) (**B**) the graphic represents the 8 CpGs sites analyzed of the promoter region of 743 patients. The dot line is the threshold of DNA methylation (>0.6 = Methylated). (**C**) Schematic representation of the *CHD5* gene that includes the promoter region and the *CHD5* first intron region analyzed by MS-PCR (153 bp PCR product) and ChIP assays (236 bp PCR product) respectively. The red arrows represent the primers employed for MS-PCR and blue arrows for ChIP qPCR (**D**) Promoter DNA methylation status was assessed by MS-PCR in MCF 10A, MCF7, MDA-MB-231 and HeLa cells. DNA from lymphocytes was methylated *in vitro* by SssI methyltransferase and used as a methylated DNA positive control (IVD). M represents methylated, and U represents non-methylated. (**E** and **F**) qPCR evaluation of the products obtained from the ChIP assay of the *CHD5* first intron, precipitated with anti-KDM4A (D), anti-H3K36me3 and anti-H3K36me2 (E) antibodies in MCF 10A, MCF7, MDA-MB-231 and HeLa cells. As a negative control, we used the IgG antibody included in the OneDay ChIP kit (Diagenode, NJ, USA, Kch-onedIP-180). (^**^) *p <* 0.01 and (^***^) *p <* 0.005 compared with the MCF 10A cell line. Statistical differences were determined using Student's *t* test.

Some authors have reported that DNA methylation at *CHD5* gene promoter can alter the expression of this gene in several cancers and neoplastic cell lines [11, 12]. Thus, we analyzed the methylation status along the *CHD5* gene locus of 743 breast cancer patients and 98 normal samples obtained from TCGA (Ilumina Human Methylation 450 K) through the TCGA wanderer web service [13]. This panel measures the methylation levels of 485,000 CpG sites distributed along the genome, of which 63 CpGs fall within the *CHD5* gene region (Figure 2A); of these sites, 8 CpGs are located within the gene promoter, the remaining 55 sites are distributed along the gene body. At the gene body, 34 CpGs are found to be methylated (having Beta-value ≥0.6, which is considered as a methylated region) in 50% of the patients, and 20 of these 34 sites that are present at the gene body are methylated in 80% of the patients. Nevertheless, when evaluating the mean methylation levels of the 8 CpG sites located within the gene promoter region (Highlighted part of the figure with a rectangle in Figure 2A) (Ensembl version 75), we observed that only 1 out of the 743 patients shows promoter methylation, where the CpG methylation Beta value is less than 0.6, indicating that *CHD5* gene promoter is considered as not methylated (Figure 2B). In order to determine if the absence of methylation in the *CHD5* promoter was restricted only to breast cancer, we also looked into the methylation status in other neoplasms such as Low-Grade Gliomas or Glioblastomas where we also did not find methylation at the promoter region (Supplementary Figure 1C). Hence, these datasets point out that DNA methylation at the promoter region is not related with *CHD5* gene silencing, suggesting that there may be other mechanisms related to its repression in breast cancer (Figure 2A).

Given the methylation status of *CHD5* gene in breast cancer patients found in TCGA, we aimed to characterize DNA methylation status at the *CHD5* promoter. We carried out a methylation sensitive-PCR assay (MS-PCR) at the CpG island which we observed to be unmethylated in 742 patients (Figure 2B and 2C). We found DNA methylation at the *CHD5* promoter to be absent in most of the cell lines, with the exception of MDA-MB-231 (Figure 2D); a similar finding was previously reported by Mulero-Navarro and Esteller [12]. As a positive methylation control of the assay we used an *in vitro*-methylated DNA (IVD) (Figure 2D).

Results from the MS-PCR reinforce the observation of the methylation status in the TCGA patients, where DNA methylation at the *CHD5* gene promoter is not a common mechanism involved in repression of *CHD5*. Therefore, we focused on another epigenetic mechanism that is independent of DNA methylation, such as the histone demethylase KDM4A.

### The localization of KDM4A at the *CHD5* first intron correlates with the decrease of H3K36me3 and H3K36me2 in neoplastic cell lines

In 2012, Mallette and colleagues demonstrated by chromatin immunoprecipitation (ChIP) assays that KDM4A is located at *CHD5* first intron in the U2OS cell line, and that the depletion of KDM4A increased *CHD5* mRNA and protein levels [7]. Nevertheless, the mechanism by which KDM4A negatively regulates transcription of the *CHD5* gene remained unclear.

One epigenetic mark relevant to transcriptional elongation is H3K36me3. This histone mark is mainly enriched in gene bodies, where a decrease in its trimethylated form is associated with gene silencing. In some genes, such silencing is not related to inactivation of the gene's promoter. Since KDM4A is capable of removing this histone mark, we speculated that demethylation of H3K36me3 could play a role in the downregulation of *CHD5* gene expression. To assess our hypothesis, we performed a ChIP assay to determine whether KDM4A could be found at the *CHD5* first intron in our cell lines. KDM4A was present at this region in the MCF7, MDA-MB-231 and HeLa cell lines; and was not detected in the non-neoplastic cell line MCF 10A (Figure 2E). One of the best-characterized gene targets of KDM4A is the region located -1922 bp upstream from the TSS of *ASCL2* [14]. Therefore, we used this region as a positive control of ChIP assay to confirm the presence of KDM4A in all the cell lines, and the 27th exon of the *RB* gene as negative control (Supplementary Figure 2A). To determine the impact of the presence of KDM4A on histone marks related to transcriptional elongation, we analyzed the abundance of H3K36me3 and H3K36me2 at the *CHD5* first intron by ChIP assay (Figure 2F). As a positive control for the H3K36me3 histone modification, we used the ENCODE database to identify a region that is enriched with this histone mark in different cell lines; based on the results of this analysis, we decided to use the second intron of the *GAPDH* gene. As a negative control, we employed the third exon of the silenced gene *MYOG* (Supplementary Figure 2B). When we compared the enrichment of the methyl marks present in the intron 1 region to the non-neoplastic cell line MCF10A we found that the presence of KDM4A was associated with a decrease in these epigenetic marks in the tumor cell lines (Figure 2F). These results suggest that the presence of KDM4A could alter epigenetic marks related to transcriptional elongation and thus affect gene transcription (Figure 2E and 2F).

### CTCF and KDM4A coexist at the *CHD5* first intron in neoplastic cell lines

**Figure 3:**
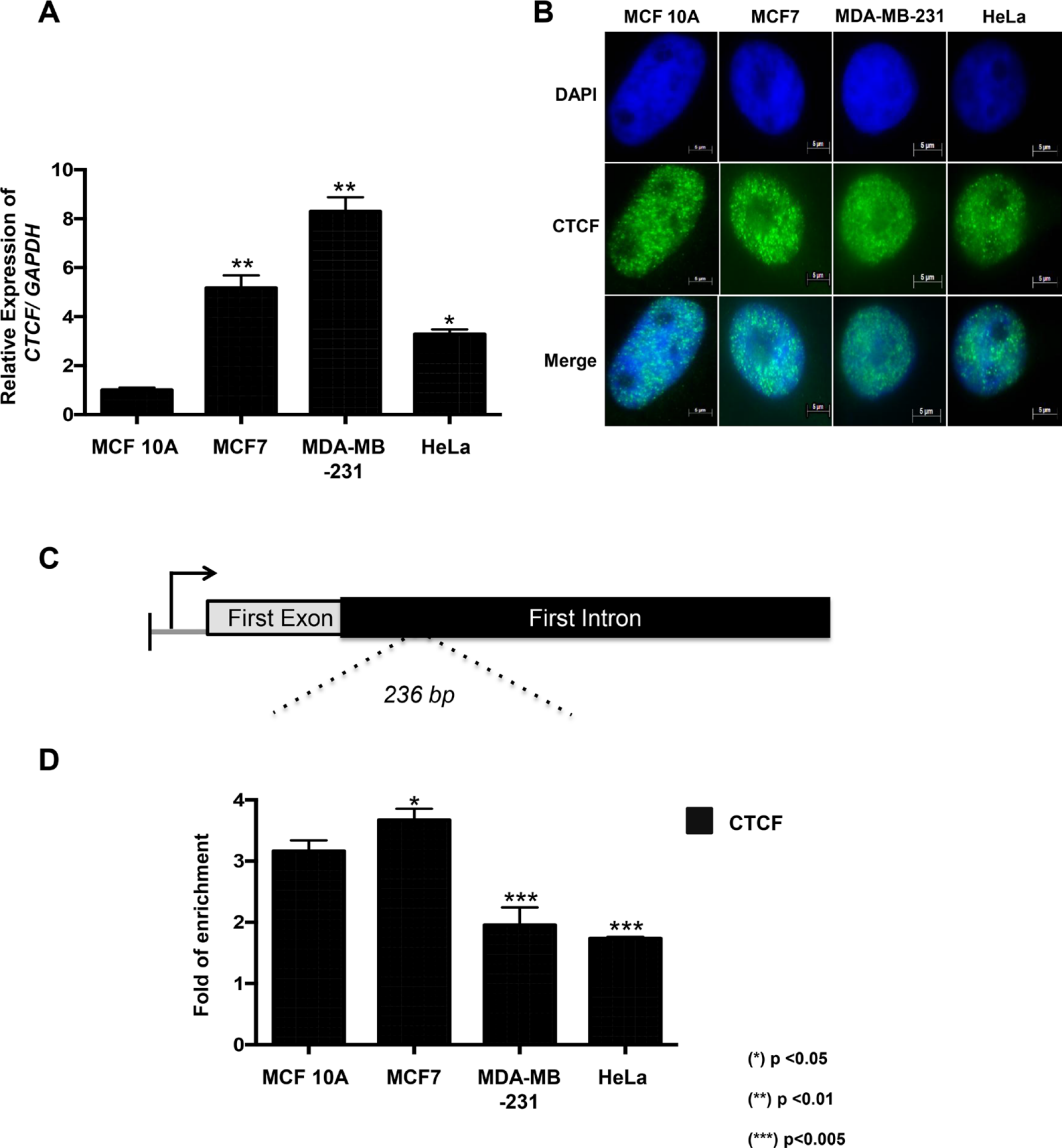
CTCF is overexpressed in neoplastic cell lines and is recruited to CHD5 first intron (**A**) Expression profile of the human *CTCF* gene in the MCF 10A, MCF7, MDA-MB-231 and HeLa cell lines was obtained with RT–qPCR. The data were normalized against GAPDH expression in three independent experiments. (^*^) *p <* 0.05 and (^**^) *p <* 0.01 compared to the MCF 10A cell line. Statistical differences were determined using Student's *t* test. (**B**) The presence and localization of CTCF in MCF 10A, MCF7, MDA-MB-231 and HeLa cells were assessed by immunofluorescence assay. (**C**) Schematic representation of the *CHD5* gene that includes the promoter region and the CHD5 first intron region (236 bp PCR product). (**D**) qPCR analysis of the *CHD5* first intron was performed on the DNA obtained from anti-CTCF ChIP assays in MCF 10A, MCF7, MDA-MB-231 and HeLa cells. As a negative control, we used the IgG antibody included in the OneDay ChIP kit (Diagenode, NJ, USA, Kch-onedIP-180). The data was evaluated by qPCR at *CHD5* first intron and the data is expressed in fold of enrichment over IgG immunoprecitpitation. Statistical differences were determined using Student's *t* test. (^*^) *p <* 0.05 and (^**^) *p <* 0.01 compared to the MCF 10A cell line.

Given that CTCF plays a major role in the demethylation function of KDM4A [8], we decided to characterize its expression in our cellular model. By RT-qPCR, we observed that CTCF was overexpressed in the MCF7, MDA-MB-231 and HeLa cell lines when compared to the MCF 10A cells (Figure 3A). In addition, CTCF was located in the nucleus of all the cell lines evaluated (Figure 3B).

**Figure 4:**
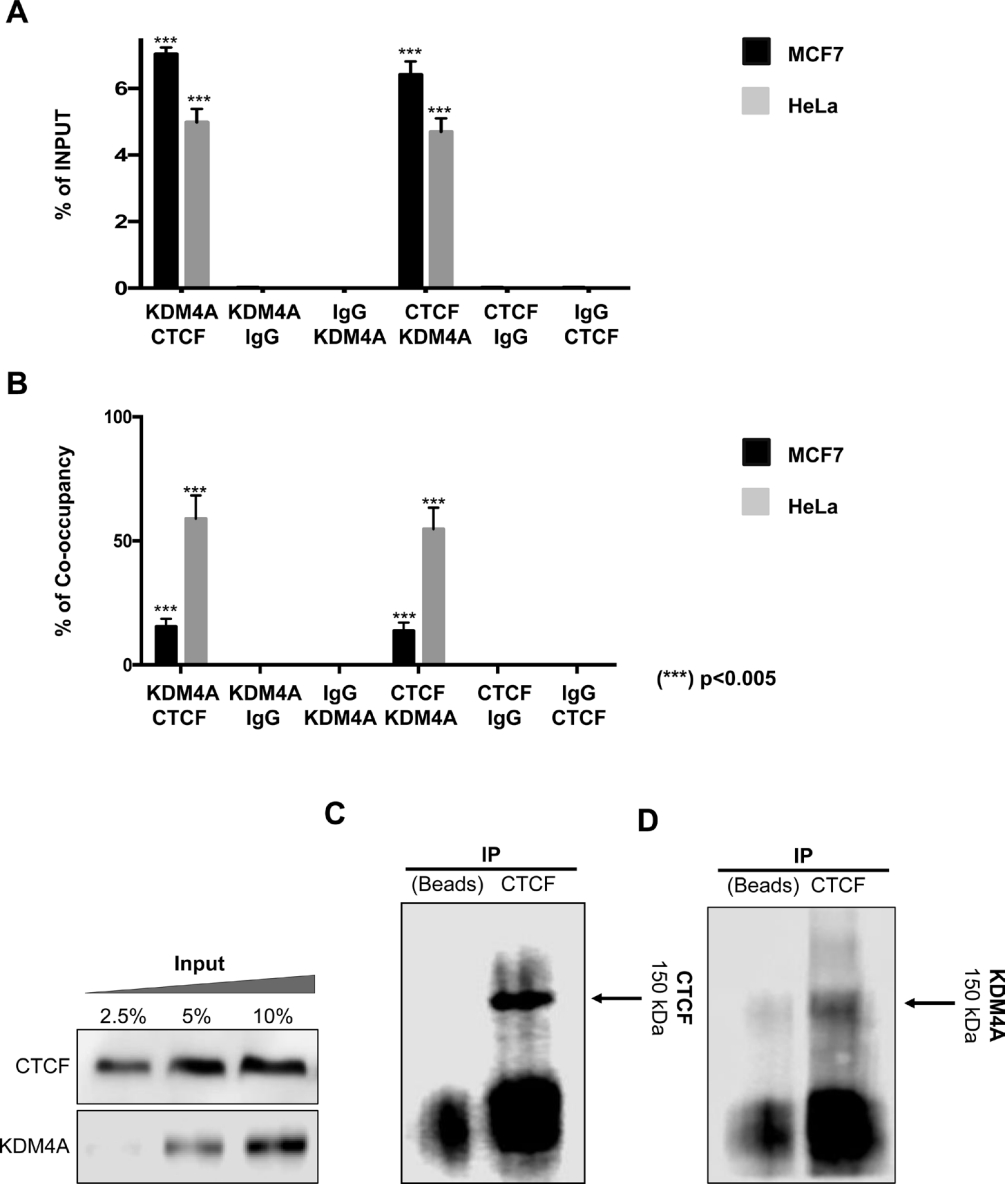
CTCF-KDM4A complex is located at the CHD5 first intron in MCF7 and HeLa cell lines (**A**) ChIP/re-ChIP assays were performed using the antibodies shown in the first row and subsequentially immunoprecipitated by the antibodies described at the second row in the MCF7 and HeLa cells. The data was evaluated by qPCR at *CHD5* first intron and the results are represented as % of input. Statistical differences were determined using Student's *t* test, (^***^) *p <* 0.005 compared with IgGs. (**B**) Co-occupancy analysis was performed in accordance to Geisberg and Struhl [38]. For occupancy analysis, the ChIP-Re-ChIP data from both experiment data (CTCF-KDM4A or KDM4A-CTCF) were used. Also, the co-occupancy of IgG experiments was evaluated. The results are represented in % of co-occupancy. Statistical differences were determined using Student's *t* test, (^***^) *p <* 0.005 compared with IgGs. (**C–D**) A Co-immunoprecipitation assay was performed against CTCF and revealed with CTCF (150 kDa) (C). Using the proteins obtained from the CTCF IP we revealed employing a KDM4A antibody (150 kDa) (D). To the left, the input material was evaluated against CTCF and KDM4A in increasing amounts of protein (2.5, 5 and 10 %).

To determine if CTCF could participate in KDM4A's demethylation activity, we decided to evaluate by ChIP assay the presence of CTCF in the first intron of *CHD5*. As a negative and positive controls, we used the 27th exon of *RB* gene and *WRAP53* promoter region, respectively (Supplementary Figure 2C). Our results show that CTCF is found in this region in all cell lines evaluated (Figure 3C and 3D), including MCF 10A. This is the same region where KDM4A was shown to be present in the neoplastic cell lines (Figure 2C). Since MDA-MB-231 exhibits promoter methylation, we decided to focus only in MCF7 and HeLa, where *CHD5* is repressed even though its promoter region is not methylated. Thus, to determine the coexistence of CTCF and KDM4A at the same genomic region, we performed a ChIP/re-ChIP experiment in the MCF7 and HeLa cell lines (Figure 4A). A first immunoprecipitation was performed with each of the antibodies (KDM4A or CTCF), and a subsequent immunoprecipitation was performed with a second antibody (KDM4A-CTCF or CTCF-KDM4A). As a negative control assay, we used the antibody of interest followed by IgGs or the IgGs followed by the antibody of interest. As a positive control for KDM4A recruitment, we analyzed the region -1922 bp from the *ASCL2* TSS (Supplementary Figure 3A). For CTCF, we employed the *WRAP53* promoter region as a positive control (Supplementary Figure 3B). As a negative control for KDM4A and CTCF, we evaluated the 27th exon of the *RB* gene (Supplementary Figure 3C). The ChIP/ReChIP results showed a co-existance of CTCF and KDM4A at the first intron of *CHD5* both in MCF7 and HeLa cells (Figure 4A). Using the ChIP and ChIP/ReChIP results we evaluated the percentage of co-occupancy in MCF7 and HeLa cell lines (Figure 4B). These results suggest that the higher co-occupancy of KDM4A and CTCF is associated with an increase in *CHD5* repression. Also, these results imply a possible interaction between CTCF and KDM4A.

### CTCF and KDM4A form a protein complex in neoplastic cell lines

In order to demonstrate the physical interaction between CTCF and KDM4A a co-immunoprecipitation assay in HeLa cells was performed. This was carried out by an immunoprecipitation against CTCF and revealed with a CTCF antibody (Figure 4C). Subsequently, the proteins obtained from the CTCF Immunoprecipitation (IP) were used in an independent experiment and were revealed against KDM4A (Figure 4D). Our data shows a detectable interaction between endogenous CTCF and endogenous KDM4A in HeLa cells (Figure 4C and 4D). Our results demonstrate a novel protein complex formed by CTCF and KDM4A, which may be localized at the first intron of the *CHD5* gene (Figure 4).

### *KDM4A* and *CTCF* siRNA knock down is associated with the reactivation of *CHD5* expression in neoplastic cell lines

**Figure 5:**
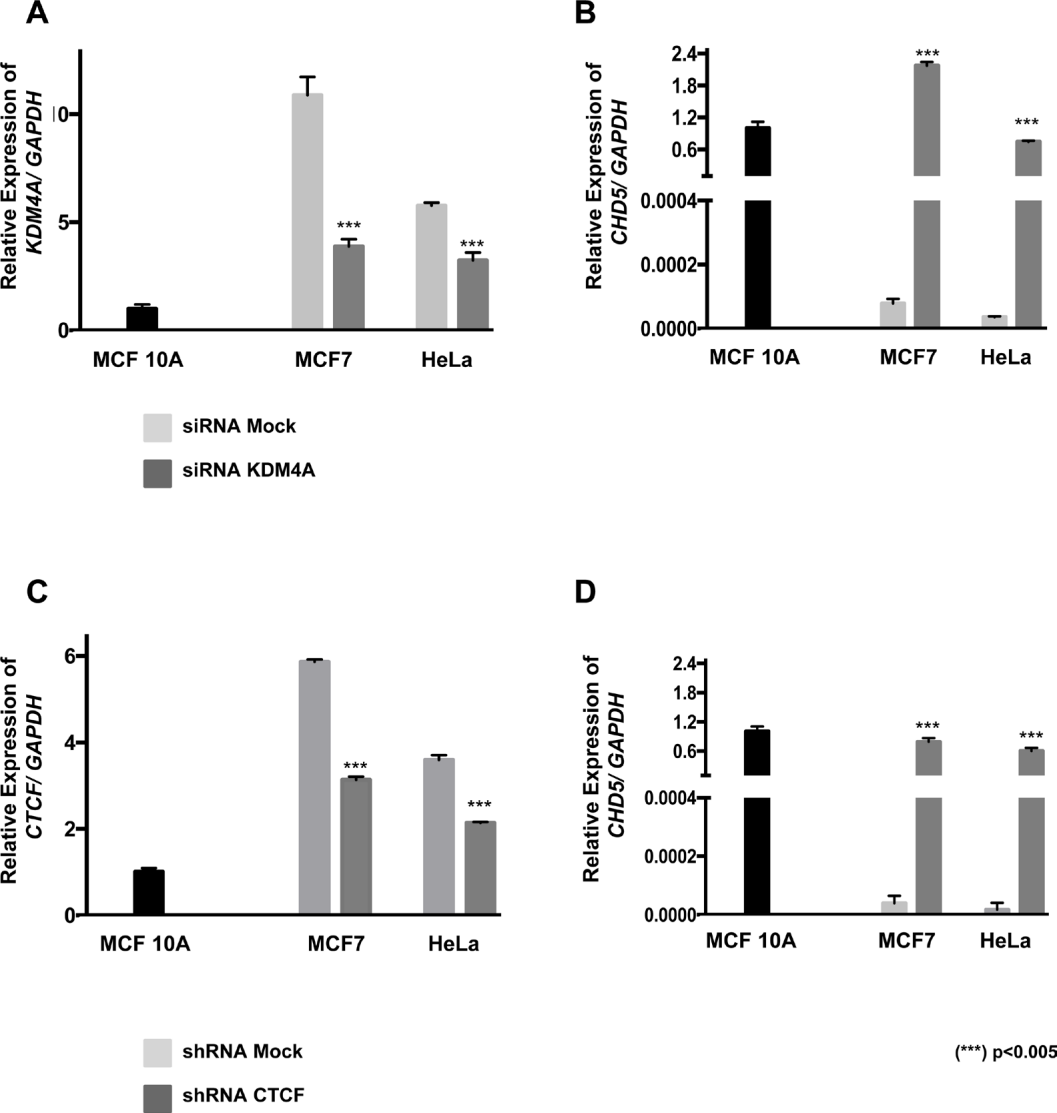
The *CHD5* expression is reactivated by CTCF and KDM4A knockdown in MCF7 and HeLa cells Analysis of *KDM4A* (**A**) and *CHD5* (**B**) expression in MCF7 and HeLa cell lines following *KDM4A* siRNA transfection. Analysis of *CTCF* (**C**) and *CHD5* (**D**) expression in MCF7 and HeLa cell lines following *CTCF* shRNA transfection. Data were normalized against *GAPDH* expression in three independent experiments using MCF 10A cells as the normal expression control. siRNA mock and shRNA mock transfected cells were used as negative controls. Statistical differences were determined using Student's *t* test compared with mock-transfected cells. (^***^) *p <* 0.005.

To determine the participation of KDM4A in the repression of *CHD5*, HeLa and MCF7 cells were transfected with siRNAs against *KDM4A*. At 72 hours, post-transfection with the siRNA, expression analyses of the *KDM4A* and *CHD5* genes were performed by RT-qPCR. The results revealed that *KDM4A* mRNA decreased after transfection (Figure 5A). The decrease of KDM4A in the MCF7 and HeLa cell lines induced the reactivation of *CHD5* mRNA, even above the basal expression of MCF 10A (Figure 5B). Because CTCF and KDM4A can potentially form a protein complex, we further investigated which was the participation of CTCF in the repression of *CHD5*. Therefore, we transient transfected a small hairpin RNA expression vector against CTCF (pCT1) in MCF7 and HeLa cells (Figure 5C). Our results show that diminishing of CTCF leads to a reactivation of *CHD5* expression similar to MCF 10A (Figure 5D). Taken together, our results suggest that the presence of KDM4A and CTCF at the first intron of *CHD5* acts as repressors of *CHD5* expression in neoplastic cells.

### *KDM4A* knockout (KO) in MCF7 reestablish the H3K36me3 histone mark at the first intron of *CHD5* and reactivates gene expression

In order to further validate that KDM4A is negatively regulating *CHD5* we establish a Knockout model (KDM4A^KO^) using CRISPR/Cas9 KO system (Santa Cruz, sc-404599 and sc-404599-HDR). This system employed three gRNAs that target exon 3 and 8 of the *KDM4A* gene (Supplementary Figure 4A). We selected cells by puromycin treatment and further enrich our KDM4A^KO^ by FACS cell sorting selecting the highest fluorescent cells (Supplementary Figure 4B). As control, we employed a non-targeting gRNA plasmid (Mock) (Santa Cruz, sc-418922).

**Figure 6:**
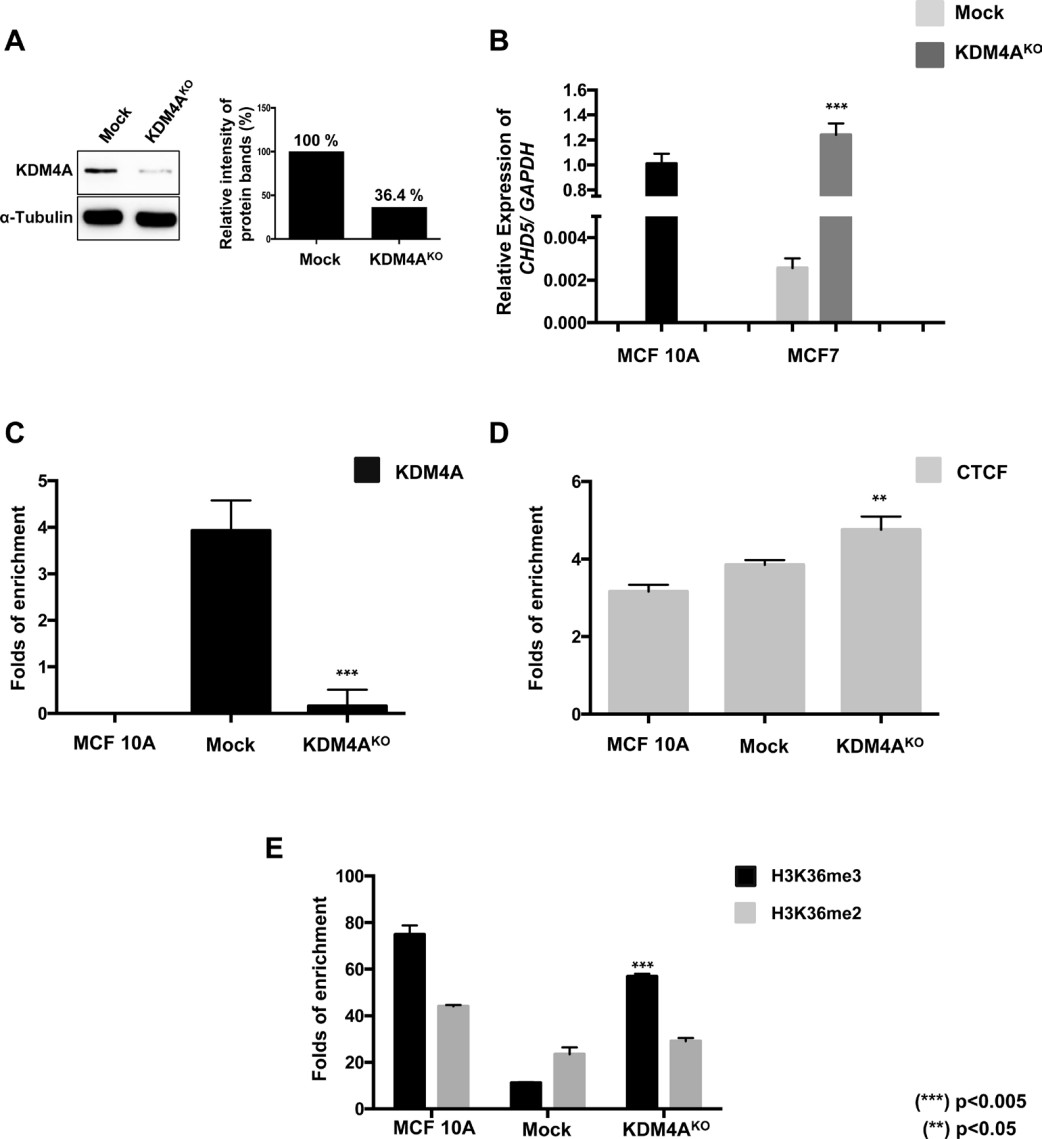
The KDM4A knockout promotes the reestablishment of the H3K36me3 histone mark at the first intron and the reactivation of the expression of CHD5 gene (**A**) Characterization of KDM4A protein abundance by immunoblots in MCF7 cells transfected with a non-targeting gRNA plasmid (Mock) or KDM4A^KO^ CRISPR/Cas9 and HDR plasmids. The quantitation of the relative intensity of the protein bands showed a decrease of 63.6% of KDM4A in KO cells. (**B**) *CHD5* expression analysis in Mock and KDM4A^KO^ cells. Data were normalized against *GAPDH* expression in two independent experiments using MCF 10A cells as the normal expression control. (**C, D, E**) qPCR evaluation of the *CHD5* first intron from DNA obtained from the ChIP assay using anti-KDM4A (C), anti-CTCF (D) and anti-H3K36me3 and anti-H3K36me2 (E) antibodies in MCF 10A cells, Mock and KDM4A^KO^ cells. As a negative control, we used the IgG antibody included in the OneDay ChIP kit (Diagenode, NJ, USA, Kch-onedIP-180). (^**^) *p <* 0.01 and (^***^) *p <* 0.005 compared with the MCF7 Mock cells.

We evaluated by Western Blot the protein expression of KDM4A in MCF7 Mock and KDM4A^KO^ cells, where a 63.6% reduction of KDM4A in KDM4A^KO^ cells is observed (Figure 6A). We also performed RT-qPCR analysis of *CHD5* expression in MCF10A, MCF7 Mock and KDM4A^KO^ cells. Here we observe a significant reactivation of the *CHD5* expression in KDM4A^KO^ cells, with levels similar to the observed in MCF10A (Figure 6B). In order to evaluate if such reactivation is related to the loss of KDM4A of the *CHD5* first intron, we performed a ChIP analysis of KDM4A. Our results show a significant loss of KDM4A in KDM4A^KO^ compared to Mock cells (Figure 6C). Regarding our previous results that suggest that KDM4A-CTCF complex regulates *CHD5*, we evaluated if CTCF could be affected by the loss of KDM4A at the *CHD5* first intron. Our results show that CTCF binding is independent of KDM4A presence, suggesting that CTCF may acts as repressor when it is in a complex with KDM4A (Figure 6D). Because of the obtained results, we attempted to establish a CTCF^KO^ model, however these cells were not viable so the experimental approach was not possible. The generation of a CTCF^KO^ model has been an experimental challenge for different research groups. Particularly, alteration in the abundance of CTCF affects cell proliferation and can even be causal of a lethal phenotype in murine models [15–17].

One of the central questions we wanted to address is whether the loss of KDM4A could restore the H3K36me3 pattern at the *CHD5* first intron. Therefore, we performed a ChIP analysis of H3K36me2/3 in MCF10A, MCF7 Mock and KDM4A^KO^ cells. We found a H3K36me3 recovery in KDM4A^KO^ cells, which does not affect the H3K36me2 (Figure 6E). This suggests that the loss of KDM4A demethylase allows the reincorporation of H3K36me3 at the first intron of *CHD5*, favoring the reactivation of the gene expression.

## DISCUSSION

Epigenetic alterations are a common feature of cancer processes [18, 19]. Mainly, key epigenetic components, which include methylases and demethylases such as KDM4A as well as architectural proteins like CTCF, are deregulated [5]. Several studies have reported that KDM4A is highly expressed in breast cancer tissues. This demethylase removes the methyl group of H3K9me3 and H3K36me3, with the former related to heterochromatin and the repression of transcription [20], while the latter is enriched in the bodies of genes that are transcriptionally active and is associated with the recruitment of RNA polymerase II and the process of transcriptional elongation [4]. Hence, H3K36me3 alteration could affect gene transcription without disturbing the gene promoters, suggesting a novel mechanism of gene dysregulation not associated with regulatory regions.

*CHD5* is a gene that encodes an enzyme which belongs to the helicase family (chromodomain helicase DNA-binding protein 5) [21]. The CHD5 protein can function as a tumor suppressor by regulating apoptosis and cellular senescence, and is involved in the p19^Arf^/p53 pathway by interacting with MDM2 [22, 23]. Because this interaction leads to the attenuation of MDM2-mediated p53 degradation [24], CHD5 and p19^ARF^ help to stabilize p53. In addition, CHD5 inhibits clonogenic growth *in vitro* as well as tumor xenograft growth, suggesting that its inactivation may be involved in cancer development [11]. Some studies have suggested that *CHD5* can be inactivated by genetic [25] or epigenetic processes, but these reports focused mainly on its repression by DNA promoter methylation [11, 12, 26–28]. Analysis of TCGA datasets show that the *CHD5* promoter region is not methylated in breast cancer patients, which suggests that another epigenetic mechanism could be involved in gene repression. In this regard there is evidence that suggests that alteration at the *CHD5* promoter is not the major mechanism of repression of this gene [12].

Previously, it was reported that KDM4A localizes to the *CHD5* first intron and the reduction in KDM4A leads to an increase of *CHD5* expression in U2O2 cells; this indicates that KDM4A could be associated with *CHD5* repression [7]. However, since KDM4A was not found at the promoter of *CHD5*, the mechanism of how KDM4A downregulates *CHD5* remained unclear.

The overexpression of *KDM4A* is known to be associated with cell proliferation and poor prognosis in several cancers [29, 30]. Identifying how KDM4A inhibits gene expression has a therapeutic impact on cancer in the future; therefore, understanding the molecular mechanisms underlying the effects of KDM4A and their implications in cancer are an important topic for future clinical research [31]. Our findings show that KDM4A functions as a repressor of the *CHD5* TSG by affecting epigenetic marks associated with elongation and not by regulating the gene promoter. This phenomenon has been reported in other cellular models, where KDM4A/C specifically alters H3K36me3 [32]. The phenomenon is also associated with the loss of RNA polymerase II recruitment in transcribed regions of the *GFAP* gene [32]. Our results suggest a novel mechanism of *CHD5* gene repression, where the decrease of H3K36me3/2 at the gene body could lead to transcriptional repression. One hypothesis is that this phenomenon occurs due to lack of phosphorylation of the second serine in the carboxy terminal domain of RNA polymerase II, which results in the enrichment of H3K36me2 and a decrease of transcriptional elongation, or due to an increase in repressive histone marks.

**Figure 7:**
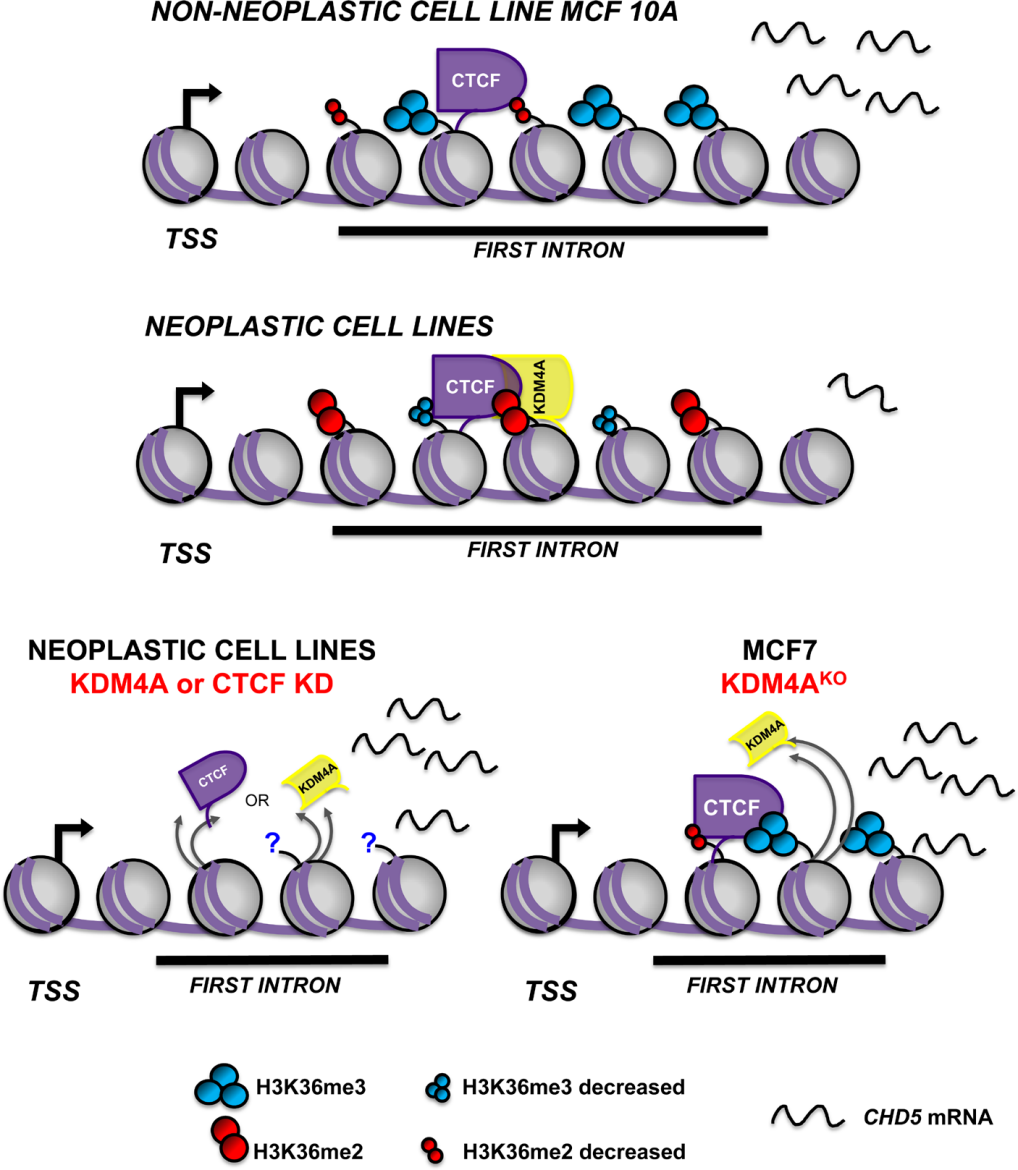
Schematic model of *CHD5* transcriptional repression mediated by CTCF-KDM4A protein complex CTCF-KDM4A protein complex is recruited to the first intron of the *CHD5* gene and promotes demethylation of histone H3K36me. In non-neoplastic cells, CTCF is located at the first intron of *CHD5*, and H3K36me3/2 are enriched. These events correlate with *CHD5* expression. In contrast, in the neoplastic cells, CTCF-KDM4A protein complex promotes the demethylation of H3K36me3/2 and leads to gene repression. CTCF or KDM4A knockdown (KD) reactivates *CHD5* gene expression. The loss of KDM4A in KDM4A^KO^ cells leads to the reestablishment of the H3K36me3 histone mark at the first intron and the reactivation of *CHD5* gene expression.

*In vitro* assays have reported that the presence of CTCF increases the demethylation frequency of KDM4A by up to 80%, suggesting that CTCF has a role in the demethylation function of KDM4A [8]. Additional support for these datasets was provided by another study that demonstrated that CTCF can interact with the KDM5B histone demethylase and increase its demethylation activity in breast cancer cell lines [33]. CTCF has been reported to act occasionally as a transcriptional repressor of genes, such as *c*-*MYC*, *Bax*, *Xist* and *hTERT*, by interacting with SIN3A and recruiting HDACs or by preventing the binding of transcription factors that affect expression [34–38]. Interestingly, we observed a protein complex formed by CTCF-KDM4A, which is found at the first intron of *CHD5*. When we evaluated the co-occupancy of KDM4A and CTCF, we showed that the HeLa cell line exhibits a higher percentage of co-occupancy in comparison with MCF7 cell line. Our results suggest that KDMA4 acts as a transcriptional repressor when it is in complex with CTCF. The loss of KDM4A at *CHD5* first intron restores H3K36me3 histone mark and recovers *CHD5* gene expression. Therefore, we propose a novel mechanism of transcriptional repression mediated by KDM4A and CTCF (Figure 7). To date it is unknown if this complex is related with the repression of other genes, and what could be the implications of this complex in diseases such as cancer. Further studies are needed to understand the biological meaning of this new regulatory mechanism.

## MATERIALS AND METHODS

### Cell culture

MCF 10A cells were cultured in 1 part DMEM-Dulbecco's Modified Eagle Medium (GIBCO, 11965-084) to 1 part of Dulbecco's Modified Eagle Medium/Ham's F-12 Nutrient Mixture (DMEM/F-12, GIBCO, 11320-033) supplemented with 10% fetal bovine serum (GIBCO, 10500056), 2 mM L-Glutamine (GIBCO, 25030081), 10 ng/Ml EGFRh (Invitrogen), 120 mU/mL insulin and 1 μg/mL hydrocortisone (SIGMA). MCF7 and MDA-MB-231 cells were cultured in Dulbecco's Modified Eagle Medium/Ham's F-12 Nutrient Mixture (DMEM/F-12, GIBCO, 11320-033) supplemented with 10% fetal bovine serum. HeLa cells were cultured in DMEM high glucose (GIBCO, 11965-084) supplemented with 10% fetal bovine serum. All cell lines were cultured at 37°C in a 5% CO_2_ incubator.

### Expression analysis

Total RNA was extracted using TRIzol (Invitrogen, 15596018) according to the manufacturer's specifications. 2 μg of total RNA were reverse transcribed in a final volume of 40 μL using the Kit GeneAmp^®^ RNA PCR KIT (Applied Biosystems, N8080143) as described by the manufacturer's protocol. Gene expression levels for *KDM4A, CTCF*, and *CHD5* were determined using the primers listed in Supplementary Table 1; *GAPDH* was used as an internal control. The qPCRs were performed using Thermo Maxima SYBR Green/ROX 1 PCR Master Mix (Thermo Scientific, K0222) with a StepOnePlus Real–Time PCR System (Applied Biosystems). All reactions were run in triplicate, and the average C_t_ values were used for quantification. The plots show the mean of three biological replicates. The analysis of the relative quantification of target genes was performed using the ΔΔC_t_ method as described by Livak [39].

### Immunofluorescence assays

Cells were cultured in 22 × 22 mm coverslips at least 18 h before the immunofluorescence staining was performed. The cells were fixed in 2% formaldehyde for 10 min and then washed three times with 1x PBS for 5 min each. Subsequently, cells were permeated with 2% Triton X-100 in 1× PBS for 20 min and then washed three times with 1× PBS for 5 min. Non-specific antigens were blocked by incubating the cells with 1% fetal bovine serum in 1× PBS for 40 min at room temperature. Then, the cells were incubated with the primary antibodies diluted in blocking solution for 60 min at 37°C. The coverslips were washed three times with 2% Triton X-100 in 1× PBS for 3 min; in between these washes, the cells were quickly rinsed with 1× PBS. Afterwards, the coverslips were incubated with the secondary antibodies diluted in blocking solution for 60 min at room temperature in the dark. The cells were washed three times with 2% Triton X-100 in 1× PBS for 3 min; in between these washes, the cells were quickly rinsed with 1× PBS. Finally, the coverslips were mounted on a previously cleaned slide with 10 μL-15 μL mounting medium with DAPI (Vector Labs, H-1200). To prevent drying and movement under the microscope, the coverslips were sealed with nail polish and then stored in the dark at 4°C. For all experiments, at least 100 cells from three coverslips were analyzed. The antibodies used are listed in Supplementary Table 2. The cells were observed using a Zeiss Axio Imager A2 epifluorescence microscope (Carl Zeiss), and the images were analyzed using AxioVision 4.8 software (Carl Zeiss). The concentrations and quantities of antibodies were chosen based on the manufacturer's specifications.

### *CHD5* Promoter methylation analysis by MS-PCR

DNA was obtained from the cell lines by phenol/chloroform extraction. 500 ng of genomic DNA were modified using the EZ DNA methylation Gold kit (ZYMO, D5006). The MS-PCR assay was performed with DNA treated with sodium bisulfite. The primers for MS-PCR were designed using Methyl Primer Express software and are listed in Supplementary Table 3. As a positive control, 1 μg of DNA from lymphocytes of a healthy donor was methylated *in vitro* (IVD) for 8 h using SssI methyltransferase (NEB, M226S).

### Chromatin immunoprecipitation (ChIP) and ChIP/re-ChIP assays

Cells were cultured until 80% confluence, and then, chromatin was extracted in accordance with the protocol of the OneDay ChIP kit (Diagenode, Kch-onedIP-180). ChIP assays were performed following the manufacturer's instructions. For all experiments, at least two chromatin preparations were analyzed. As a negative control, we used an IgG antibody included in the kit. The antibodies used are listed in Supplementary Table 4.

The ChIP/re-ChIP assays were performed following the method previously described by [40]. In brief, cells were treated according to the first steps in the ChIP assay and then incubated at 37°C in 10 mM DTT in 1X ChIP buffer for 30 min. Eluents were then diluted at 1.5 mL with ChIP buffer and incubated with the indicated second antibody overnight. The following day, protein A agarose beads were added to the solution, which was then incubated for 3 h at 4°C. The DNA-protein-antibody complexes were washed three times with 1X ChIP buffer. Finally, the DNA-protein complexes were treated with proteinase K overnight, and to break the crosslinked complexes, the samples were boiled for 10 min. The DNA was extracted as suggested by the OneDay ChIP kit protocol, and qPCR was performed with the specific primers listed in Supplementary Table 5.

The obtained results represent experiments from four separate amplifications that were used to calculate the standard deviation. qPCRs were done in triplicate using fast optical 96-well qPCR plates. Then, the oligonucleotides were amplified in triplicate by a fast optical 96-well qPCR plate (Applied Biosystems). The qPCR was performed using Thermo Maxima SYBR Green/ROX 1 PCR Master Mix (Thermo Scientific, K0222) with a StepOnePlus Real–Time PCR System (Applied Biosystems). We used the concentration of antibodies indicated by the manufacturer's specifications.

### ChIP and ChIP-Re-ChIP data analysis

The oligonucleotides were validated with a standard curve performed with Input serial dilutions. The amplification efficiency (AE) value was calculated as AE = 10^(−1/slope). The percentage of the input was calculated as % input = AE^(Ctinput – CtChIP) × Fd (Fd = factor Dilution) × 100, using 10% of the input value as reference. Afterwards, to calculate the fold of enrichment of the immunoprecipitated proteins we used the following equation fold of enrichment = % input (ip)/% input (IgG) as described in the OneDay ChIP (Diagenode) manufacturer's manual. For the ChIP/Re-ChIP analysis, we calculated the % of the input using the 10% of the input as reference and compared the data obtained from IgG.

### Co-occupancy data analysis

To determine the co-occupancy of CTCF and KDM4A at the first intron of *CHD5* gene, we used the fold of enrichment over the background for each individual ChIP. The percentage of co-occupancy, was calculated according to Geisberg and Struhl [41]: % co-occupancy = 100 (AB–A)/(A × B–A), where A and B represent the IP of each experiment, and AB the ChIP-Re-ChIP assay. The occupancy was determined in the ChIP-Re-ChIP data for both experiments (CTCF-KDM4A or KDM4A-CTCF), as well as the co-occupancy in the IgG experiments, with negative results plotted with a value of 0.

### CTCF and KDM4A knockdown

HeLa and MCF7 cells were transient transfected using Xfect transfection reagent (Clontech, 631317) following the manufacturer's specifications, using 2.5 μg of an small hairpin RNA expression vector against CTCF (pCT1) kindly provided by Ko Ishihara (Institute of Molecular Embriology and Genetics, Kumamoto University, Japan) [42]. As a mock control, we employed the empty vector from pSilencer-3.1-H1 puro (Ambion).

For KDM4A knockdown, siRNA transfections were performed using KDM4A SMART pool siRNAs (Dharmacon, E-004292-00-0010) and non-targeting siRNA (Dharmacon, D-001910-01-05). HeLa and MCF7 cells were seeded at 3 × 10^4^ cells/well and 6 × 10^4^ cells/well, respectively, in 12-well plates. 24 h later, the cells were transfected with ACCELL siRNA Delivery Media (Dharmacon, B-005000-500) over 72 h according to the manufacturer's protocol. The results were obtained from three separate biological replicates. RNA and cDNA were obtained as previously described.

### Co-immunoprecipitation of CTCF and KDM4A (Co-IP)

Extracts from HeLa cells were prepared with IP lysis buffer containing 50 mM Tris-HCl (pH 8.0), NaCl 150 mM and 1% of NP40 supplemented with 2× complete protease inhibitor cocktail (Sigma-Aldrich). The cell lysate was cleared by centrifugation at 13,000 rpm for 10 minutes at 4°C. The proteins were incubated with 2 μg of anti-CTCF (Santa Cruz Biotech, sc-5916) or without antibody (using beads) and the complex were precipitated employing 25 μL of Protein A/G magnetic beads (Pierce, 88802) and incubated at 4°C approximately 16 h. The beads were recovered with a magnetic stand, and washed five times for 20 minutes with IP lysis buffer. Finally, proteins were eluted by boiling in 1× Laemmli buffer and evaluated by Western Blot using antibodies against CTCF (Santa Cruz Biotech, sc-5916) and KDM4A (Cell Signaling, JMJD2A #5328) as two independent experiments. At least three independent biological replicates were evaluated.

### CRISPR/Cas9 KO and HDR plasmids transfection

We used the X-fect Transfection Reagent (PT5003-2) to transfect 1 μg of the CRISPR/Cas9 KDM4A KO Plasmid (sc-404599) and 1 μg of the HDR Plasmid (sc-404599-HDR). In brief, 3 × 10^5^ cells were seeded in a 6-well chamber, 24 h before plasmid transfection. We diluted 1 μg of the KDM4A KO and HDR plasmids onto 100 μL of Xfect Reaction Buffer. Afterwards, we added 2 μL of the Xfect Polymer and incubated for 15 min. Finally, we distributed the entire 100 μL of nanoparticle complex solution dropwise to the cell culture medium. 48 h after transfection we evaluate the GFP and RFP expression by epifluorescence microscopy (Carl Zeiss, AXIO Imager D2). We selected the transfected cells with media supplemented with Puromycin (3 μg/mL), changing the media every 24 h for at least 5 days.

### Flow cytometry and cell sorting

KDM4A^KO^ or Mock cells were resuspended at a concentration of 1 × 10^6^ cells/mL in DMEM/F-12, containing 10% FBS and 1X antibiotic-antimycotic. First, cells were filtered through a 70 μm cell strainer and subsequently through a 40 μm cell strainer and sorted on a FACSAria III Cell Sorting Flow Cytometer (BD Biosciences, San Jose, CA). Prior to sorting, MCF7 WT was used for cell size and autoflorescence measures. It was determined that the Mock cells did not show RFP florescence, while the KO population that was positive for RFP, only cells with the highest fluorescence were sorted (Supplementary Figure 4B). 2.61 × 10^5^ cells were sorted into DMEM/F12 medium containing 10% FBS, and 2X antibiotic-antimycotic and were seeded in a p60 cell culture plate. The KDM4A^KO^ population, that exhibit RFP+ high expression, and Mock sorted cells were used for the subsequent experiments.

## SUPPLEMENTARY MATERIALS FIGURES AND TABLES



## Abbreviations

KDM4A: Lysine specific demethylase 4
CTCF: CCCTC-Binding Factor
CHD5: chromodomain helicase DNA binding protein 5
qPCR: quantitative PCR
ChIP: Chromatin immunoprecipitation
Co-IP: co-immunoprecipitation
MS-PCR: Methylation-specific PCR
H3K36me3: trimethylation of histone H3 at lysine 36
H3K36me2: demethylation of histone H3 at lysine 36
TSG: tumor suppressor gene

## References

[1] Kouzarides T (2007). Chromatin modifications and their function. Cell.

[2] Martin C, Zhang Y (2005). The diverse functions of histone lysine methylation. Nat Rev Mol Cell Biol.

[3] Guerra-Calderas L, González-Barrios R, Herrera LA, Cantú de León D, Soto-Reyes E (2015). The role of the histone demethylase KDM4A in cancer. Cancer Genet.

[4] Pradeepa MM, Sutherland HG, Ule J, Grimes GR, Bickmore WA (2012). Psip1/Ledgf p52 binds methylated histone H3K36 and splicing factors and contributes to the regulation of alternative splicing. PLoS Genet.

[5] Berry WL, Shin S, Lightfoot SA, Janknecht R (2012). Oncogenic features of the JMJD2A histone demethylase in breast cancer. Int J Oncol.

[6] Kolla V, Zhuang T, Higashi M, Naraparaju K, Brodeur GM (2014). Role of CHD5 in human cancers: 10 years later. Cancer Res.

[7] Mallette FA, Richard S (2012). JMJD2A promotes cellular transformation by blocking cellular senescence through transcriptional repression of the tumor suppressor CHD5. Cell Reports.

[8] Jeong YS, Park JS, Ko Y, Kang YK (2011). JHDM3A module as an effector molecule in guide-directed modification of target chromatin. J Biol Chem.

[9] Zhang D, Yoon HG, Wong J (2005). JMJD2A is a novel N-CoR-interacting protein and is involved in repression of the human transcription factor achaete scute-like homologue 2 (ASCL2/Hash2). Mol Cell Biol.

[10] Barretina J, Caponigro G, Stransky N, Venkatesan K, Margolin AA, Kim S, Wilson CJ, Lehár J, Kryukov GV, Sonkin D, Reddy A, Liu M, Murray L (2012). The Cancer Cell Line Encyclopedia enables predictive modelling of anticancer drug sensitivity. Nature.

[11] Du Z, Li L, Huang X, Jin J, Huang S, Zhang Q, Tao Q (2016). The epigenetic modifier CHD5 functions as a novel tumor suppressor for renal cell carcinoma and is predominantly inactivated by promoter CpG methylation. Oncotarget.

[12] Mulero-Navarro S, Esteller M (2008). Chromatin remodeling factor CHD5 is silenced by promoter CpG island hypermethylation in human cancer. Epigenetics.

[13] Díez-Villanueva A, Mallona I, Peinado MA (2015). Wanderer, an interactive viewer to explore DNA methylation and gene expression data in human cancer. Epigenetics Chromatin.

[14] Tan MK, Lim HJ, Harper JW (2011). SCF(FBXO22) regulates histone H3 lysine 9 and 36 methylation levels by targeting histone demethylase KDM4A for ubiquitin-mediated proteasomal degradation. Mol Cell Biol.

[15] Moore JM, Rabaia NA, Smith LE, Fagerlie S, Gurley K, Loukinov D, Disteche CM, Collins SJ, Kemp CJ, Lobanenkov VV, Filippova GN (2012). Loss of maternal CTCF is associated with peri-implantation lethality of Ctcf null embryos. PLoS One.

[16] González-Buendía E, Pérez-Molina R, Ayala-Ortega E, Guerrero G, Recillas-Targa F (2014). Experimental strategies to manipulate the cellular levels of the multifunctional factor CTCF. Methods Mol Biol.

[17] Splinter E, Heath H, Kooren J, Palstra RJ, Klous P, Grosveld F, Galjart N, de Laat W (2006). CTCF mediates long-range chromatin looping and local histone modification in the beta-globin locus. Genes Dev.

[18] Cloos PA, Christensen J, Agger K, Helin K (2008). Erasing the methyl mark: histone demethylases at the center of cellular differentiation and disease. Genes Dev.

[19] Jovanovic J, Rønneberg JA, Tost J, Kristensen V (2010). The epigenetics of breast cancer. Mol Oncol.

[20] Rea S, Eisenhaber F, O'Carroll D, Strahl BD, Sun ZW, Schmid M, Opravil S, Mechtler K, Ponting CP, Allis CD, Jenuwein T (2000). Regulation of chromatin structure by site-specific histone H3 methyltransferases. Nature.

[21] Thompson PM, Gotoh T, Kok M, White PS, Brodeur GM (2003). CHD5, a new member of the chromodomain gene family, is preferentially expressed in the nervous system. Oncogene.

[22] Serrano M, Lin AW, McCurrach ME, Beach D, Lowe SW (1997). Oncogenic ras provokes premature cell senescence associated with accumulation of p53 and p16INK4a. Cell.

[23] Bagchi A, Papazoglu C, Wu Y, Capurso D, Brodt M, Francis D, Bredel M, Vogel H, Mills AA (2007). CHD5 is a tumor suppressor at human 1p36. Cell.

[24] Tao W, Levine AJ (1999). P19(ARF) stabilizes p53 by blocking nucleo-cytoplasmic shuttling of Mdm2. Proc Natl Acad Sci USA.

[25] Fujita T, Igarashi J, Okawa ER, Gotoh T, Manne J, Kolla V, Kim J, Zhao H, Pawel BR, London WB, Maris JM, White PS, Brodeur GM (2008). CHD5, a tumor suppressor gene deleted from 1p36.31 in neuroblastomas. J Natl Cancer Inst.

[26] Fatemi M, Paul TA, Brodeur GM, Shokrani B, Brim H, Ashktorab H (2014). Epigenetic silencing of CHD5, a novel tumor-suppressor gene, occurs in early colorectal cancer stages. Cancer.

[27] Zhao R, Yan Q, Lv J, Huang H, Zheng W, Zhang B, Ma W (2012). CHD5, a tumor suppressor that is epigenetically silenced in lung cancer. Lung Cancer.

[28] Mokarram P, Kumar K, Brim H, Naghibalhossaini F, Saberi-firoozi M, Nouraie M, Green R, Lee E, Smoot DT, Ashktorab H (2009). Distinct high-profile methylated genes in colorectal cancer. PLoS One.

[29] Hu CE, Liu YC, Zhang HD, Huang GJ (2014). JMJD2A predicts prognosis and regulates cell growth in human gastric cancer. Biochem Biophys Res Commun.

[30] Wang B, Fan X, Ma C, Lei H, Long Q, Chai Y (2016). Downregulation of KDM4A Suppresses the Survival of Glioma Cells by Promoting Autophagy. J Mol Neurosci.

[31] Franci G, Sarno F, Nebbioso A, Altucci L (2017). Identification and characterization of PKF118-310 as a KDM4A inhibitor. Epigenetics.

[32] Cascante A, Klum S, Biswas M, Antolin-Fontes B, Barnabé-Heider F, Hermanson O (2014). Gene-specific methylation control of H3K9 and H3K36 on neurotrophic BDNF versus astroglial GFAP genes by KDM4A/C regulates neural stem cell differentiation. J Mol Biol.

[33] Yamamoto S, Wu Z, Russnes HG, Takagi S, Peluffo G, Vaske C, Zhao X, Moen Vollan HK, Maruyama R, Ekram MB, Sun H, Kim JH, Carver K (2014). JARID1B is a luminal lineage-driving oncogene in breast cancer. Cancer Cell.

[34] Filippova GN, Fagerlie S, Klenova EM, Myers C, Dehner Y, Goodwin G, Neiman PE, Collins SJ, Lobanenkov VV (1996). An exceptionally conserved transcriptional repressor, CTCF, employs different combinations of zinc fingers to bind diverged promoter sequences of avian and mammalian c-myc oncogenes. Mol Cell Biol.

[35] Méndez-Catalá CF, Gretton S, Vostrov A, Pugacheva E, Farrar D, Ito Y, Docquier F, Kita GX, Murrell A, Lobanenkov V, Klenova E (2013). A novel mechanism for CTCF in the epigenetic regulation of Bax in breast cancer cells. Neoplasia.

[36] Sun S, Del Rosario BC, Szanto A, Ogawa Y, Jeon Y, Lee JT (2013). Jpx RNA activates Xist by evicting CTCF. Cell.

[37] Renaud S, Loukinov D, Bosman FT, Lobanenkov V, Benhattar J (2005). CTCF binds the proximal exonic region of hTERT and inhibits its transcription. Nucleic Acids Res.

[38] Lutz M, Burke LJ, Barreto G, Goeman F, Greb H, Arnold R, Schultheiss H, Brehm A, Kouzarides T, Lobanenkov V, Renkawitz R (2000). Transcriptional repression by the insulator protein CTCF involves histone deacetylases. Nucleic Acids Res.

[39] Livak KJ, Schmittgen TD (2001). Analysis of relative gene expression data using real-time quantitative PCR and the 2(-Delta Delta C(T)) Method. Methods.

[40] Truax AD, Greer SF (2012). ChIP and Re-ChIP assays: investigating interactions between regulatory proteins, histone modifications, and the DNA sequences to which they bind. Methods Mol Biol.

[41] Geisberg JV, Struhl K (2005). Analysis of protein co-occupancy by quantitative sequential chromatin immunoprecipitation. Curr Protoc Mol Biol.

[42] Ishihara K, Oshimura M, Nakao M (2006). CTCF-dependent chromatin insulator is linked to epigenetic remodeling. Mol Cell.

