# Supplementary material for: CTCF-KDM4A complex correlates with histone modifications that negatively regulate CHD5 gene expression in cancer cell lines
Source: Oncotarget. 2018 Mar 30;9(24):17028–42. doi: 10.18632/oncotarget.24798 (PMC5908303; doi:10.18632/oncotarget.24798)
Supplement: Supplementary file 1 [file oncotarget-09-17028-s001.pdf]

CTCF-KDM4A complex correlates with histone modifications that negatively regulate *CHD5* gene expression in cancer cell lines

SUPPLEMENTARY MATERIALS

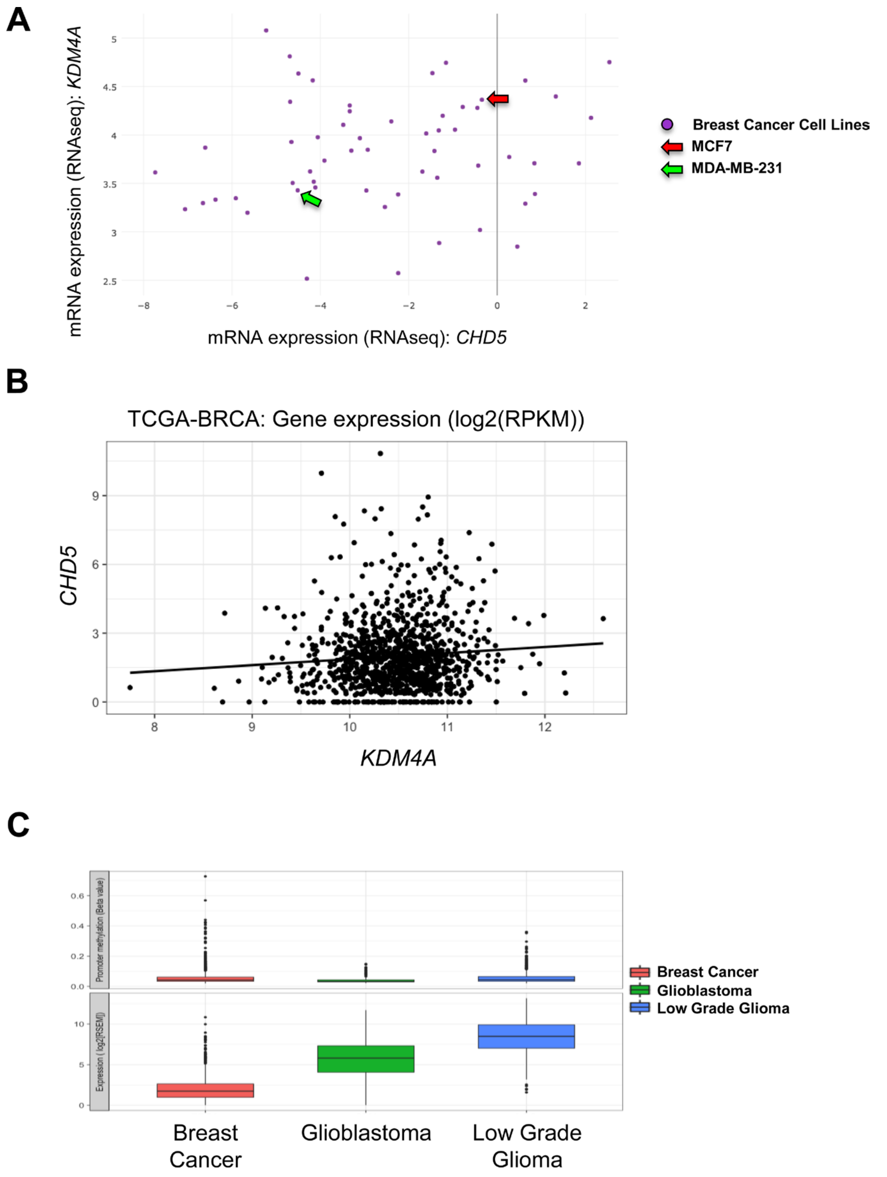

Supplementary Figure 1: *In silico* analysis of *KDM4A* and *CHD5* expression by the Cancer Cell Line Encyclopedia and TCGA databases. (**A**) Expression of *CHD5* vs *KDM4A* in multiple breast cancer cell lines available at the cancer cell line encyclopedia. Purple circles represent the expression of each breast cancer cell line. The red and green arrows point MCF7 and MDA-MB-231 cell lines [10]. (**B**) RNA-seq gene expression of *CHD5* vs *KDM4A* in TCGA breast cancer patients. (**C**) Promoter methylation and expression levels of *CHD5* in TCGA Breast Cancer, Glioblastoma and Low Grade Glioma, plot produced by [43].

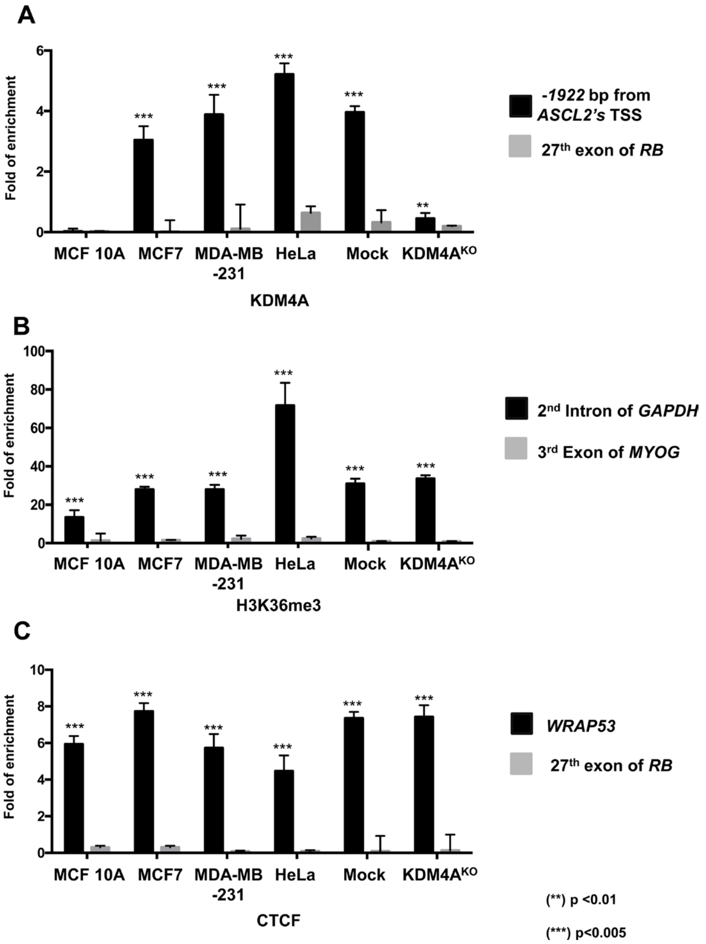

Supplementary Figure 2: Controls for KDM4A, H3K36me3 and CTCF immunoprecipitations. (**A**) ChIP assay against KDM4A, evaluated by qPCR in MCF 10A, MCF7, MDA-MB-231 and HeLa cells. As a positive control for KDM4A enrichment, we used the region located at -1922 bp from the TSS of the *ASLC2* gene. As a negative control, we employed the 27th exon of RB gene. (**B**) Control for H3K36me3 immunoprecipitation. ChIP assay against H3K36me3, evaluated by qPCR in MCF 10A, MCF7, MDA-MB-231 and HeLa cells. As a positive control for H3K36me3, we used the second intron of the *GAPDH* gene (grey), and as a negative control, we used the third exon of the *MYOG* gene (black). (**C**) Control for CTCF immunoprecipitation. ChIP assay against CTCF, evaluated by qPCR in MCF 10A, MCF7, MDA-MB-231 and HeLa cells as a positive control for CTCF enrichment, we used the promoter of the WRAP53 gene. As a negative control, we employed the 27th exon of RB gene. We used the IgG antibody included in the OneDay ChIP kit (Diagenode, NJ, USA, Kch-onedIP-180), as negative control, (**) $p < 0.01$, and (***) $p < 0.005$ compared with immunoprecipitation of the IgG. Statistical differences were determined using Student's *t* test.

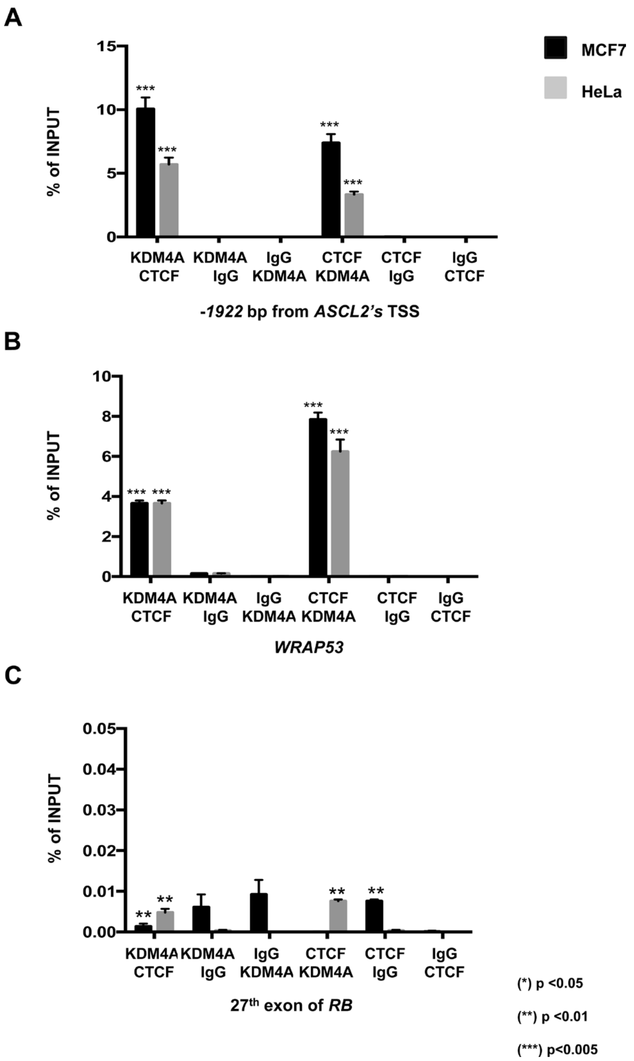

Supplementary Figure 3: Controls for KDM4A and CTCF ChIP/re-ChIP. (**A** and **B**) ChIP/re-ChIP assay against KDM4A and CTCF evaluated at the positive control regions located -1922 bp from the TSS of the *ASLC2*gene (A) and the *WRAP53* gene promoter (B) as a positive control for CTCF and as a negative control, we employed the 27th exon of the *RB* (**C**) gene in MCF7 and HeLa cells. The first IPs were performed with the antibodies shown in the first row and were followed by the IPs described in the second row. (*) $p < 0.05$, (**) $p < 0.01$, and (***) $p < 0.005$ compared with immunoprecipitation of the IgG. Statistical differences were determined using Student's *t* test.

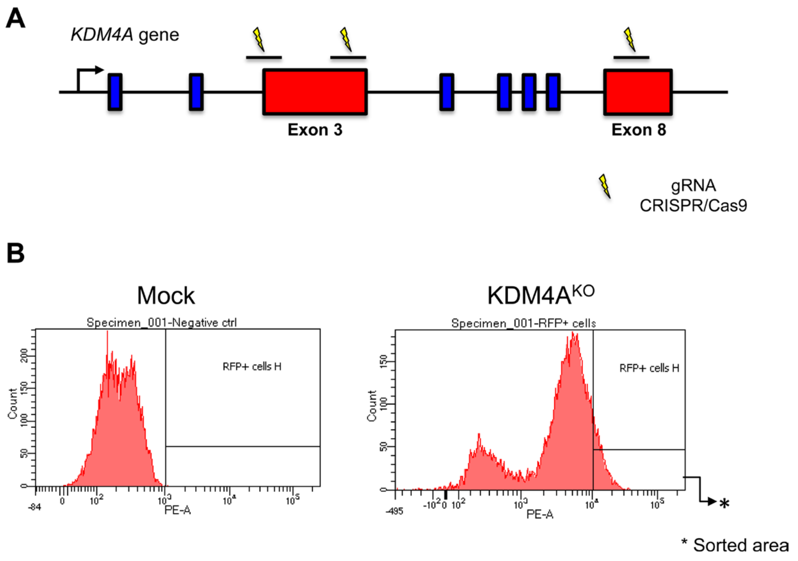

Supplementary Figure 4: Flow cytometry and cell sorting (**A**) KDM4A^{KO} cells were generated by CRISPR/Cas9 KO system. This system employed three gRNAs that targets exon 3 and exon 8 for *KDM4A* gene. As Mock control a non-targeting gRNA plasmid was used. (**B**) The KDM4A^{KO} cells were selected by puromycin and RFP fluorescence was determined in Mock and KDM4A^{KO} cells. The KDM4A^{KO} cells with the highest fluorescent cells were sorted (*defines the sorted area).

Supplementary Table 1: Primers list for expression analysis

Gene amplified	Primers	DNA sequence (5′-3′)	Amplicon length in cDNA
KDM4A	KDM4A FWD	CGGCCAAGTCTATGGAGCC	*177 bp*
	KDM4A RVS	TCATTGAAGCGCATGTCTGAG	
CHD5	CHD5 FWD	TCGAGACTTCCCTGTGTTGC	*127 bp*
	CHD5 RVS	CTTTTTGTCCCAAGGTGGCG	
CTCF	CTCF FWD	GAGAAGCCATTCAAGTGTTCCAT	*85 bp*
	CTCF RVS	CTCCAGTATGAGAGCGAATGTGA	
GAPDH	GAPDH FWD	TGCACCACCAACTGCTTAGC	*87 bp*
	GAPDH RVS	GGCATGGACTGTGGTCATGAG	

Supplementary Table 2: Antibodies used for the immunofluorescence assays

TARGET	KDM4A	CTCF	CHD5
CAT. NUMBER	pAb-126-050	sc-398149	sc-68389
MANUFACTURER	Diagenode	Santa Cruz BT	Santa Cruz BT

Supplementary Table 3: Primers list for MS-PCR

Type of oligo	Primers	DNA sequence (5′-3′)	Amplicon length
METHYLATED	PROM CHD5 M FWD	TTGTGCGTTGTGATCGTC	*153 bp*
	PROM CHD5 M RVS	AAACGTCAAACCCGTAACC	
UNMETHYLATED	PROM CHD5 UN FWD	TTTTTGTGTGTTGTGATTGTT	*153 bp*
	PROM CHD5 UN RVS	AAACATCAAACCCATAACCAAA	

Supplementary Table 4: Antibodies used for the ChIP assay

TARGET	KDM4A	CTCF	H3K36me3	H3K36me2
CAT. NUMBER	ab105953	07-729	C15410058	CS-127-100
MANUFACTURER	Abcam	Millipore	Diagenode	Diagenode

Supplementary Table 5: Primers list used for the ChIP analysis

Amplified region	Primers	DNA sequence (5′–3′)	Amplicon length
+741 bp in CHD5	KDM4A in CHD5 FWD	TCCCAAGCACTTTACCCG	*236 bp*
	KDM4A in CHD5 RVS	AAGAACTGTCCCGCAAGG	
2ND intron in GAPDH	+ Ctrol K36me3 FWD	GATGCTGAGTGTACAAGCGTTTT	*172 bp*
	+ Ctrol K36me3 RVS	AGTCATACGAAGCCCTTCCA	
3RD exon in MYOG	–Ctrol K36me3 FWD	GGCCACAGATGCCACTACTT	*204 bp*
	–Ctrol K36me3 RVS	GCTTTACCTCCCTGGAAAGG	
-1922 bp in ASCL2	+ Ctrol KDM4A FWD	CGAAGACCGGGTAGAGAG	*80 bp*
	+ Ctrol KDM4A RVS	TCTGGGTGTGGCTGCATAC	
WRAP53 promoter	+ Ctrol CTCF FWD	GACAGGTCTGAAGCCTG	*230 bp*
	+ Ctrol CTCF RVS	CGGGACGTGAAAGGTTAG	
27th Exon in RB	–Ctrol CTCF FWD	CTAACACTGGCATGTTCAAAGC	*163 bp*
	–Ctrol CTCF RVS	GGTGTAGGGGAGGGG	

List of primers used in this study for expression, MS-PCRs and ChIP assays, and list of antibodies used in immunofluorescences and ChIPs experiments.

REFERENCES

43. Tang Z, Li C, Kang B, Gao G, Li C, Zhang Z. GEPIA: a web server for cancer and normal gene expression profiling and interactive analyses. Nucleic Acids Res. 2017; 45:W98–102. https://doi.org/10.1093/nar/gkx247.
